# Task-Free Functional MRI in Cervical Dystonia Reveals Multi-Network Changes That Partially Normalize with Botulinum Toxin

**DOI:** 10.1371/journal.pone.0062877

**Published:** 2013-05-01

**Authors:** Cathérine C. S. Delnooz, Jaco W. Pasman, Christian F. Beckmann, Bart P. C. van de Warrenburg

**Affiliations:** 1 Radboud University Medical Centre, Donders Institute for Brain, Cognition and Behaviour, Department of Neurology, Nijmegen, The Netherlands; 2 MIRA Institute, Twente University, Enschede, The Netherlands; 3 Radboud University, Donders Institute for Brain, Cognition and Behaviour, Centre for Cognitive Neuroimaging, Nijmegen, The Netherlands; University of British Columbia, Canada

## Abstract

Cervical dystonia is characterized by involuntary, abnormal movements and postures of the head and neck. Current views on its pathophysiology, such as faulty sensorimotor integration and impaired motor planning, are largely based on studies of focal hand dystonia. Using resting state fMRI, we explored whether cervical dystonia patients have altered functional brain connectivity compared to healthy controls, by investigating 10 resting state networks. Scans were repeated immediately before and some weeks after botulinum toxin injections to see whether connectivity abnormalities were restored. We here show that cervical dystonia patients have *reduced* connectivity in selected regions of the prefrontal cortex, premotor cortex and superior parietal lobule within a distributed network that comprises the premotor cortex, supplementary motor area, primary sensorimotor cortex, and secondary somatosensory cortex (sensorimotor network). With regard to a network originating from the occipital cortex (primary visual network), selected regions in the prefrontal and premotor cortex, superior parietal lobule, and middle temporal gyrus areas have reduced connectivity. In selected regions of the prefrontal, premotor, primary motor and early visual cortex *increased* connectivity was found within a network that comprises the prefrontal cortex including the anterior cingulate cortex and parietal cortex (executive control network). Botulinum toxin treatment resulted in a partial restoration of connectivity abnormalities in the sensorimotor and primary visual network. These findings demonstrate the involvement of multiple neural networks in cervical dystonia. The reduced connectivity within the sensorimotor and primary visual networks may provide the neural substrate to expect defective motor planning and disturbed spatial cognition. Increased connectivity within the executive control network suggests excessive attentional control and while this may be a primary trait, perhaps contributing to abnormal motor control, this may alternatively serve a compensatory function in order to reduce the consequences of the motor planning defect inflicted by the other network abnormalities.

## Introduction

Cervical dystonia (CD) is the most common type of primary dystonia. [1] It is characterized by involuntary, abnormal movements and postures of the head. Injecting the involved cervical muscles with botulinum toxin (BoNT) is an effective and evidence-based treatment of CD and leads to marked improvement in head posture and secondary symptoms such as pain. Patients with dystonia have abnormal motor system physiology, as for example shown by the loss of surround inhibition, that leads to unnecessary contractions of more muscles than required for given motor behaviour. [2], [3] Other studies have also indicated abnormalities in somatosensory processing and sensorimotor integration in patients with dystonia [4]–[9], possibly as a consequence of underlying maladaptive synaptic plasticity [10], [11], of which the lack of inhibition could again be the driver. [12].

Current theories on the pathophysiology of dystonia are largely based on studies in focal hand dystonia, and in particular writer’s cramp (WC). Despite some clinical overlap and electrophysiological similarities, the pathophysiology of CD is likely to be, at least in part, different from that of WC, for example given the lack of task-specificity in CD. The few functional MRI and PET studies conducted in CD patients have mainly used event-related paradigms, which makes it difficult to conclude whether the observed effects represent the primary pathophysiological mechanism, a secondary (feedback) mechanism, or a compensatory effect to a specific task. [5], [6], [13], [14].

Functional MRI (fMRI) is a powerful tool to assess disease-related cerebral activity. Over the recent years, the evaluation of functional relationships across distributed brain regions by means of resting state functional connectivity, has turned out to be a valuable instrument with which further insight into the cerebral mechanisms that underlie various neurological diseases, including dystonia, can be obtained. [15]–[17] Functional connectivity is represented by intrinsic temporal interactions between various brain regions. It provides an index that exploits physiological fluctuations in the blood oxygen level dependent (BOLD) signal and is thought to reflect the hemodynamic consequences of variations in transient neuronal dynamics that propagate through anatomically connected networks. [18], [19] To further clarify primary changes in functional connectivity, one can apply a network analysis, based on independent component analysis (ICA), on BOLD time series obtained with the use of resting state fMRI, i.e. fMRI acquired in the absence of an explicit goal-directed task. ICA extracts spatiotemporal patterns of underlying signal components, including BOLD resting state networks (RSN), assuming that the components are statistically independent. [20] It has been shown that several important RSNs, such as the sensorimotor network (SMN) and default mode network (DMN), can be obtained with high reliability across individuals and studies. [20]–[22].

In order to gain more insight in the pathophysiology of CD, we investigated functional connectivity with the use of resting state fMRI in CD patients and healthy controls. We hypothesized to find changes in four networks: the SMN, cerebellar network (CN), and DMN, based on previous work by others and ourselves [16], [17], [23], and also the executive control network (ECN), which is not only involved in motor control but also the non-motor symptoms that are frequently found in dystonia, e.g mood disorders, pain and executive dysfunction. [24] Lastly, to test the hypothesis that BoNT treatment could modify connectivity in affected RSNs, we evaluated resting state connectivity in a paired pre- and post-treatment design.

## Methods

### Ethics Statement

Subjects were included after providing written informed consent. Both the study and consent procedure were approved by the Medical Review Ethics Committee region Arnhem-Nijmegen.

### Subjects

Twenty-three CD patients (9 men; mean age 57.2 years, range 40–82 years) and 22 healthy controls (10 men; mean age 54.5 years, range 41–72 years) were included. Patients were excluded if aged under 18 years, when showing severe head tremor or dystonia outside the cervical region, and when not receiving regular BoNT treatment. Torticollis was the dominant feature in 16 patients, of which 9 patients presented with torticollis to the left. In the other patients, laterocollis was the predominant symptom. (Table S1) Patients were all treated with BoNT type A (Dysport®) every two to four months with objective and subjective clinical success (mean duration BoNT: 7.6 years); no other neurotropic medication was used. The Toronto Western Spasmodic Torticollis Rating Scale (TWSTRS) [25] severity subscore was used to measure dystonia severity. (Table S1) The results for TWSTRS scores ranged from 6 to 22 (mean 18.4) before injection (t = 0) and from 1 to 17 (mean 10.4) after injection (t = 1), demonstrating a significant improvement due to BoNT (TWSTRS, Z = −4.11, p = .00).

### Image Acquisition

For the longitudinal aspect of this study, subjects were scanned before BoNT injection (t = 0), after 4–5 weeks (t = 1), and directly before the next (t = 2) BoNT treatment. The timing of t = 1 was chosen because a maximal effect of the BoNT injections could be expected at this point. The mean delay between BoNT treatment and the MRI scan was 93.2±13.9 days for the t = 0 scan and 93.2±17.9 days for the t = 2 scan (Interval t = 0–t = 2, Z = −.21, p = .83). Subjects were instructed to lie still (i.e. in complete supine position, eyes closed) during the fMRI scan, to avoid sleep, and not to think of something in particular. This was confirmed in a post-scanning debriefing. Head movements were minimized by an adjustable padded head holder. Images were acquired on a 3-T Siemens Magnetom Allegra Scanner (Erlangen, Germany) equipped with a 32-channel head coil using multi-echo scanning. The multi-echo scan comprised acquisition of four echoes starting with the shortest possible TE: using GRAPPA [26] parallel imaging with an acceleration factor 3 (30 reference lines for weight set calculation acquired at start of scan); this gives echo times ranging from 6.6 to 44 ms (TR 2000 ms, FOV 224 mm^2^, voxel size 3.5×3.5×3.0 mm^3^). To allow the use of standard fMRI preprocessing tools, the four echoes were combined using the procedure described in Poser et al. While multi-echo acquisition improves the BOLD contrast-to-noise ratio (CNR) by covering a larger range of T^*^_2_’s, the optimum contrast for different brain regions will be spread over the different TE images. To optimize the sensitivity gain from the broadened T^*^_2_ coverage we used pixelwise weighted echo summation based on CNR measurements. [27] High-resolution anatomical images were acquired using an MP-RAGE sequence (TE/TR 3.03/2300 ms, 192 sagittal slices, voxel size 1.0×1.0×1.0 mm^3^, FOV 256 mm^2^, GRAPPA with acceleration factor 2 and 50 reference lines).

### FMRI Analysis

#### Data pre-processing

Pre-processing consisted of motion correction, removal of non-brain structures, spatial smoothing using a Gaussian kernel of full width at half maximum of 5 mm, grand-mean intensity normalisation of the entire 4D dataset by a single multiplicative factor; high-pass temporal filtering (Gaussian-weighted least-squares straight line fitting, with sigma = 50.0 s) using FEAT (FMRI Expert Analysis Tool) Version 5.98, part of FSL (FMRIB’s Software Library, www.fmrib.ox.ac.uk/fsl). [28] FMRI volumes were registered to the individual’s structural scan using FMRIB’s Linear Image Registration Tool (FLIRT) and to MNI-152 standard space images using FMRIB’s Nonlinear Image Registration Tool (FNIRT). Pre-processed functional data containing 260 time points for each subject, after discarding the first five time points, were temporally concatenated across subjects to create a single 4D data set.

#### Statistical analysis

FMRI analysis was carried out using a regression technique (dual regression) that allows for voxel-wise comparisons of resting functional connectivity. [29] This approach proceeds in two stages. First, the dual-regression approach is used to identify subject-specific temporal dynamics and associated spatial maps within each subject’s fMRI data set. This first involves performing a multiple linear regression analysis on a set of PICA (Probabilistic Independent Component Analysis) spatial maps of healthy subjects derived from Smith et al. [22] against the separate fMRI data sets, resulting in matrices describing temporal dynamics for each component and subject. These time course matrices are then used in a second multiple linear regression analysis against the associated fMRI data set to estimate subject-specific spatial maps. These final spatial maps characterize the subject- and voxel specific degree of connectivity within a given component map. Subsequently, the different component maps are collected across subjects into single 4D files (one per original component map, with the fourth dimension being subject identification).

Between-group effects were assessed non-parametrically using FSL’s Randomize tool, Version 2.8 [30], incorporating threshold-free cluster enhancement (TFCE). [31] Statistics were derived for the contrasts reflecting the between-group effects (patients versus controls) and statistical significance was assessed by performing 5000 random permutations and testing the difference between groups for each iteration (controls vs. patients’ t = 0). In order to assess pre- versus post-treatment differences in the patient group (t = 0 vs. t = 1 vs. t = 2), a natural extension of the paired t-test (tripled t-test) was used including 21 patients (one patient did not complete the full experimental procedure due to non-dystonia-related reasons; the functional images (t = 1 and t = 2) of one patient could not be used due to imaging artefacts). In both designs, covariates for age and gender were added to control for possible age- and gender-related connectivity differences. Statistical results were corrected for family-wise erros (FWE) across space using cluster correction [31] and Bonferroni correction across the four different networks of interest (p≤0.013; SMN, DMN, CN and ECN). We were also able to explore six other RSNs: the medial visual, primary visual, lateral visual, auditory network and, left and right frontoparietal network (FPN). [22] In an exploratory evaluation of these non-hypothesized networks, we applied a less conservative approach, thresholding statistical maps at p≤0.05 corrected for FWE. In order to verify that the previous stated effects were consistent over time, we performed a second independent t-test evaluating differences between patients’ t = 2 and controls (data not shown).

Possible correlations between functional connectivity and disease severity (TWSTRS scores)/duration were assessed by multiple regression correcting for age, gender and GM volume in a separate analysis using the method described above. In addition, we performed a separate Spearman correlation using SPSS 20 aimed at exploring correlations between treatment-related connectivity and clinical treatment effect, while adjusting for age and gender. The clinical treatment effect was defined as the difference in TWSTRS scores for t = 0 vs. t = 1 and t = 2 vs. t = 1. Subject-specific connectivity measures were extracted from those regions that showed significant and consistent changes in treatment-related connectivity, i.e. the left and right ventral premotor cortex (see results).

### Gray Matter Morphology

Cervical dystonia-related gray matter (GM) abnormalities have been found in several brain regions though results have been inconsistent. [32]–[36] To exclude that altered functional connectivity was explained by differences in GM volume, a subject-specific voxel-dependent GM regressor and a GM mask were added to the statistical design. That is, at every voxel in the analysis, the subject-specific GM probability was used as a confound regressor. In this way we avoid estimating differences in functional connectivity that can be explained purely by differences in GM morphology. To retrieve subject-specific GM maps, all structural images were first brain extracted, then tissue-type segmented using FSL’s FAST 4.1, normalized to MNI-152 standard space and non-linearly registered. These GM maps were then used to calculate an average GM map, which was used to mask each subject-specific RSN. Also, each subject-specific GM map was added as voxel-dependent explanatory variable in the statistical model described above.

## Results

### Hypothesis-based RSN Analysis

**Figure 1:**
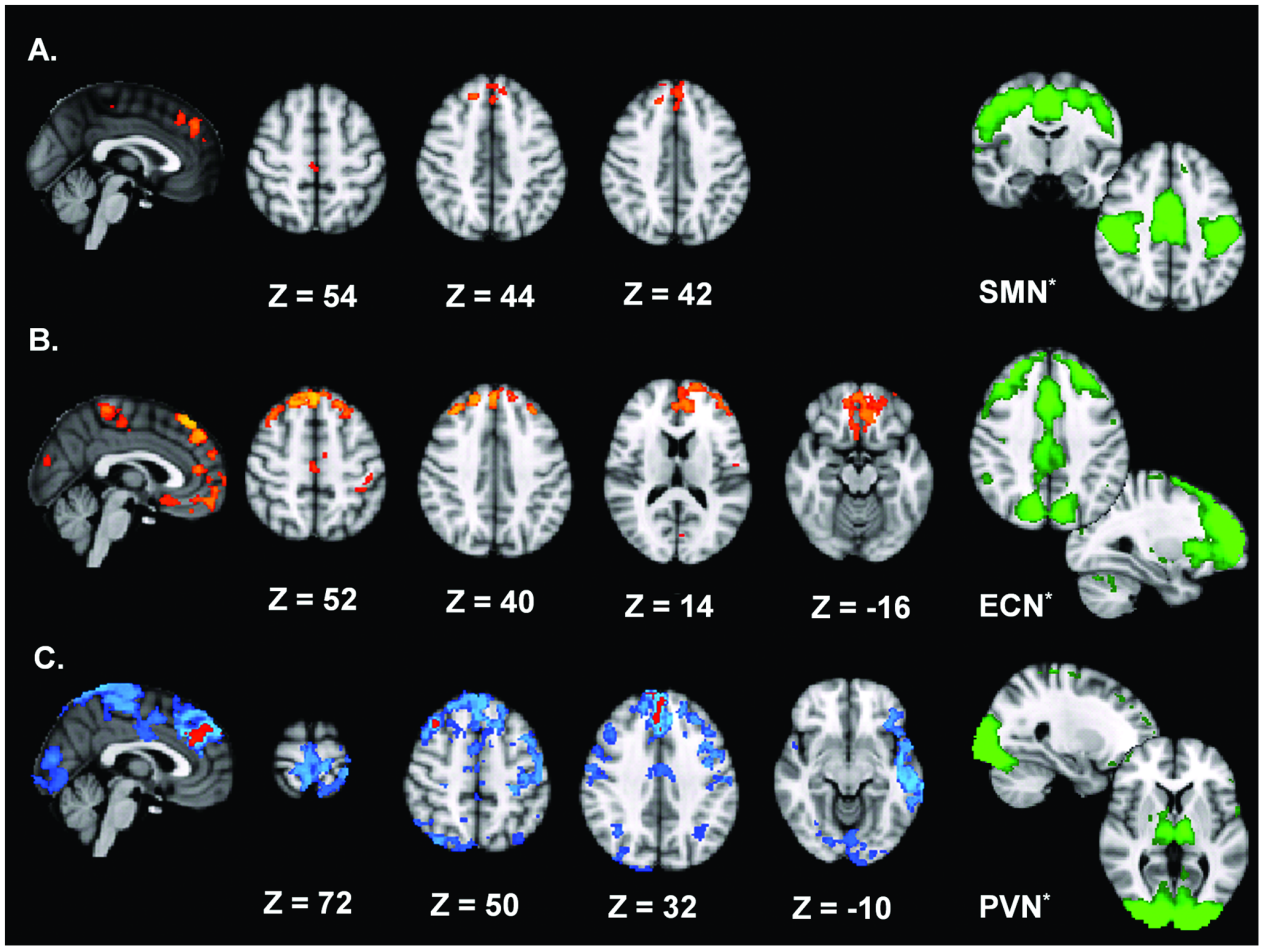
Between-group effects for the SMN, ECN and PVN. Depicted here are the between-group effects for three RSNs. Between-group effects are corrected for family-wise errors (p≤0.013 for **A.** and **B.;** p≤0.05 (blue) and p≤0.013 (orange) for **C.**). **A.** shows frontal regions and precentral regions abnormally connected to the *SMN*, indicating *de*creased connectivity within the CD group. **B.** shows brain regions linked to the *ECN*, exhibiting *in*creased connectivity for the CD group. **C.** The *PVN* shows CD-related *de*creased connectivity of several regions including PFC, PMC, SM1, and visual and temporal areas. *The right column (green) shows the original RSNs used in the dual regression approach, thresholded at Z = 2,0. These are PICA spatial maps of healthy subjects derived from Smith et al. [22] Images are t-statistics overlaid on the MNI-152 standard brain. The left hemisphere of the brain corresponds to the right side in this image.

Between-group differences in the spatial distribution of the functional connectivity maps were found in two RSNs. The first network consisted of an assembly of functionally connected regions in the prefrontal cortex (PFC), premotor cortex (PMC), primary sensorimotor cortex (SM1), secondary somatosensory cortex including the superior parietal lobule, i.e. the sensorimotor network. For CD patients, *decreased* connectivity within this network was found for regions in the bilateral PFC, bilateral PMC, and left superior parietal lobule (SPL). (Table 1, Figure 1A) At a less stringent inference (p≤0.05 FWE corrected), also SM1 and the extrastriate cortex demonstrated a similar decrease in connectivity. (Table S2, figure S1) In contrast to the SMN, CD patients exhibited increased connectivity with regard to the ECN, which consists of functionally connected regions in the frontal cortex including the anterior cingulate and paracingulate, and parietal cortex. Several regions in the bilateral superior frontal and medial gyrus, bilateral middle frontal gyrus, bilateral paracingulate gyrus and rectal gyrus, and medial paracentral lobule, corresponding to the PFC, PMC and parts of the primary sensorimotor cortex [37], and regions in the early visual cortex demonstrated increased connectivity within the ECN. (Table 1, Figure 1B) At a less stringent inference (p≤0.05 FWE corrected), also an extension of SM1, the SPL, and the extrastriate cortex demonstrated a similar increase in connectivity. (Table S3, figure S1) No significant differences were found for the CN or DMN. The analysis of controls versus patients’ t = 2 confirmed the patterns of altered connectivity (data not shown).

**Table 1:** Local maxima of regions with altered connectivity within the ECN and SMN.

RSN	Contrast	Region	Area	Side	X	Y	Z	p-value	Cluster size (voxels)
**Executive control network**	**P_t = 0_> C**	Superior medial gyrus	8	Left	0	32	54	0.003	4353
		Superior frontal gyrus	6	Right	16	34	56	0.004	
			8/9		18	44	42	0.004	
			6	Left	−14	28	56	0.004	
			9/10		−20	60	6	0.006	
		Middle frontal gyrus	8/9	Right	36	36	36	0.004	
		Mid orbital gyrus	11	Right	6	56	−2	0.006	
		Anterior cingulate cortex	25/32	Right	4	44	12	0.006	
				Left	−8	44	12	0.005	
		Rectal gyrus	11	Left	−10	46	−16	0.010	
		Paracentral lobule	4/6	Left	−6	−32	62	0.005	450
		Middle cingulate cortex	6	Left	0	−14	44	0.006	
		Postcentral gyrus	2/3b	Left	−44	−26	48	0.006	150
			OP4		−54	−10	14	0.010	6
		Calcarine gyrus	V1	Left	−6	−84	6	0.010	53
		Cuneus	V2	Left	4	−84	20	0.010	26
**Sensori**-**motor network**	**C >P_t = 0_**	Superior frontal gyrus	8/9	Left	−2	46	36	0.005	761
			8/9	Right	6	62	22	0.012	
		Superior medial gyrus	8/9	Right	14	44	38	0.008	
		Anterior cingulate cortex	25/32	Right	6	46	28	0.012	
		SMA	6	Right	4	−24	54	0.008	100
		Precuneus	5 M	Left	−4	−42	56	0.012	2

C = controls, OP = operculum, P = patients, RSN = resting state network, SMA = supplementary motor area. Between-group effects are corrected for family-wise errors (p*≤*0.013).

No correlation was found between functional connectivity and disease characteristics.

### Exploratory RSN Analysis

We explored six further RSNs, derived from Smith et al. [22]: left and right frontoparietal network, the auditory network, and three visual networks, applying a less stringent inference of p≤0.05 FWE-corrected. Only the primary visual network (PVN), originating from the occipital cortex, demonstrated differential connectivity. Between-group analysis showed decreased connectivity in CD patients for selected regions in the bilateral PFC, PMC, SM1, SPL, paracingulate gyrus, several parts of the visual cortex, and left middle temporal gyrus. (Table 2, Figure 1C.).

**Table 2:** Local maxima of regions with altered connectivity within the PVN.

RSN	Contrast	Region	Area	Side	X	Y	Z	p-value	Cluster size (voxels)
**Primary visual network**	**C >P_ t = 0_**	Anterior cingulate cortex	25/32	Left	2	34	26	0.009	21466
		Superior frontal gyrus	6	Right	20	4	56	0.039	
		Middle frontal gyrus	8/9	Right	38	22	48	0.010	
		Inferior frontal gyrus	45/44	Left	−54	30	−8	0.049	
				Right	60	22	8	0.049	
		Superior medial gyrus	8/9	Left	0	44	40	0.010	
		Middle temporal gyrus	21	Left	−58	−14	−20	0.016	
		Precentral gyrus	6	Left	−40	−14	62	0.017	
			4p/3b		−54	−4	28	0.034	
		Paracentral lobule	6	Right	6	−22	−76	0.016	
		Postcentral gyrus	3b/6/2/1	Left	−22	−34	66	0.017	
			4a/p	Left	−50	−8	38	0.049	
			2/1	Right	60	−18	38	0.034	
		Middle occipital gyrus	V3v/V2	Right	36	−96	4	0.032	
			V2	Left	−26	102	0	0.049	
		Calcarine gyrus	V1	Left	2	−100	−4	0.032	
		Lingual gyrus	V4/V3v	Left	−32	−90	−16	0.032	
			V4	Right	34	−70	−20	0.032	
		Inferior parietal lobule	PGp/a	Left	−34	−74	48	0.035	390
		Angular gyrus	hIP1/2/3	Left	−36	−60	34	0.040	
		Superior parietal lobule	7A	Left	−20	−64	68	0.048	

C = controls, P = patients, RSN = resting state network. Between-group effects are corrected for family-wise errors (p≤0.05).

Although not reaching significance, we would like to mention the result of increased connectivity of the right mid-posterior putamen within the left FPN, at an inference of p≤0.05 uncorrected, as this might be important for future connectivity studies in dystonia.

### Treatment-related Connectivity

**Figure 2:**
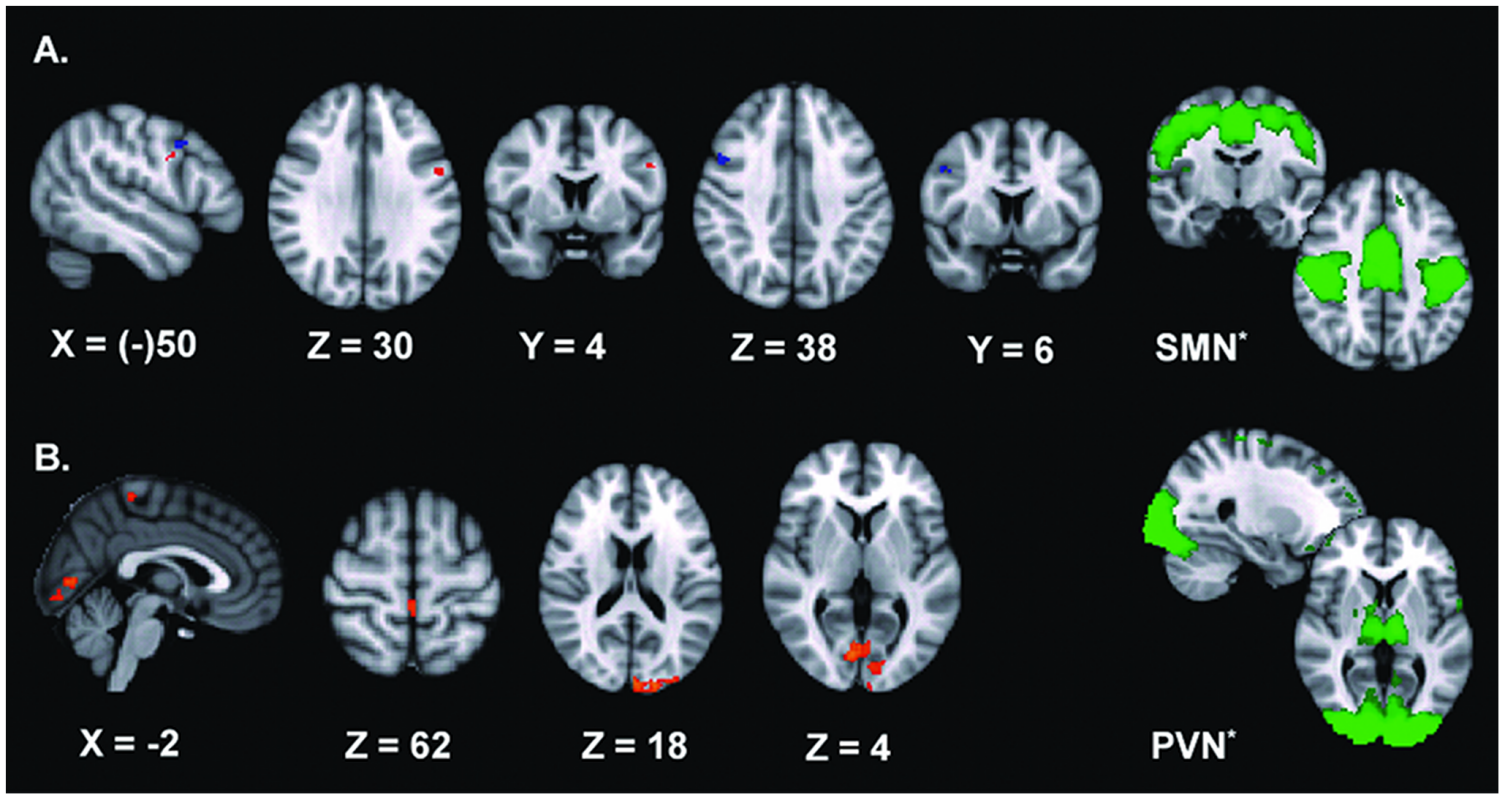
Treatment-related effects for the SMN and PVN. Depicted here are the treatment-related effects for two RSNs, corrected for family-wise errors (p≤0.05). **A.** shows the ventral premotor cortex abnormally connected to the *SMN*, but with an increase of connectivity after BoNT treatment (in orange t = 1>t = 0, in blue t = 1> t = 2). In the sagittal plane, the effect for t = 1> t = 2 (blue) is projected on the left hemisphere for graphical purposes. **B.** shows areas in the visual cortex and primary motor cortex linked to the *PVN*, also exhibiting increased connectivity after BoNT treatment. ^*^The right column (green) shows the original RSNs used in the dual regression approach, thresholded at Z = 2,0. These are PICA spatial maps of healthy subjects derived from Smith et al. [22] Images are t-statistics overlaid on the MNI-152 standard brain. The left hemisphere of the brain corresponds to the right side in this image.

We compared the connectivity maps from all three timepoints, evaluating BoNT-driven connectivity changes. There were indeed changes for the SMN and PVN, but both not surviving stringent Bonferonni correction. With regard to the SMN, increased connectivity was found after BoNT treatment (t = 0> t = 1) in the left medial frontal gyrus, corresponding to the ventral premotor cortex [38]; a similar effect was seen for t = 1> t = 2 but contralaterally. Bilateral V2, and medial M1 [37] demonstrated increased connectivity within the PVN after BoNT injections. (Table 3, Figure 2) No significant differences were found between the t = 0 and t = 2 scans. (data not shown) No correlations were found between functional connectivity and clinical treatment effect.

**Table 3:** Local maxima of regions with altered connectivity in relation to BoNT treatment.

RSN	Contrast	Region	Area	Side	X	Y	Z	p-value	Cluster size (voxels)
**Sensori-motor network**	**t = 1> t = 0**	Precental gyrus	6 (PMv)	Left	−52	4	30	0.055	94
	**t = 1> t = 2**	Precental gyrus	6 (PMv)	Right	50	6	38	0.044	16
		Middle occipital gyrus	V5	Left	−42	−76	2	0.041	16
**Primary visual network**	**t = 1> t = 0**	Superior occipital gyrus	V2	Left	−20	−94	22	0.023	679
			V5	Left	−14	−84	−14	0.041	81
		Inferior occipital gyrus	V5	Left	−42	−70	−12	0.040	20
		Postcentral gyrus	4	Left	0	−36	62	0.034	20

PMv = ventral premotor cortex, RSN = resting state network. Between-group effects are corrected for family-wise errors (p≤0.05).

## Discussion

In this study of CD, we explored alterations in whole brain functional connectivity and the possible modifying effect of BoNT injections on these alterations, by means of repetitive resting state fMRI. We found evidence for altered functional connectivity within three RSNs: the sensorimotor network, the executive control network and the primary visual network. We also collected evidence that these alterations are partially restored by BoNT injections, but these data need to be confirmed. Below, we will try to put these observations into a pathophysiological framework in the context of CD.

### Hypothesis-based RSN Analysis – Sensorimotor Network

Within the SMN, which is primarily concerned with motor planning, motor execution and sensory processing, the PFC, PMC and SPL demonstrated *decreased* connectivity in CD patients compared to healthy controls. After BoNT treatment, a relative increase of connectivity within CD patients was found for the ventral premotor cortex, partially restoring dystonia-related connectivity abnormalities.

This cluster of regions and related pathways that are abnormally connected within the SMN, are key areas in motor control. [39]–[42] Whereas the PFC plays a critical role in the selection of relevant stimuli (attentional selection) while building a motor representation and is important in the control engaged to stop or override motor responses (motor inhibition) [43], the PMC exerts a more direct influence on motor output during motor behaviour. The loss of connectivity between the SMN and the aforementioned regions may reflect an alteration in synaptic activity of the premotor-parietal and prefontal-parietal circuits, resulting in a defect of integrative motor planning. An electrophysiological correlate for faulty motor preparation was previously found in CD patients. [9], [44] We ourselves have recently demonstrated reduced integrity of the functional premotor-parietal circuitry in WC with a resting state fMRI study. [17] These results suggest that in (focal) dystonia patients there is faulty processing of motor programs, possibly as part of a larger planning defect, resulting in the difficulty centring a motor command onto the appropriate muscles.

### Hypothesis-based RSN Analysis – Executive Control Network

In contrast to the PVN and SMN, we found *increased* functional connectivity within the ECN in CD patients. Regions in this network are involved in several cognitive tasks, e.g. working memory, attention, and motor planning. [43] CD patients had increased connectivity in the PFC, PMC, SM1, and the early visual cortex with regard to the ECN. At a less stringent inference (p<0.05 FWE corrected), also SPL and extrastriate cortex demonstrated a similar increase in connectivity.

Loss of connectivity of several premotor and prefrontal regions within the SMN eluded to above, may be the functional imaging correlate of abnormal motor planning. Since the ECN is also engaged in these processes, we are tempted to hypothesize that the increase of connectivity of regions involved in motor planning and execution, within the ECN serves a compensatory role, with the attempt to regain control over the motor planning process. In other words, the dystonic brain reorganizes by creating stronger coupling to regions involved in the ECN. This increased attentional control of motor behaviour is known to improve task performance [45]–[47] and may perhaps here be used to try and alleviate dystonic symptoms. In accordance to our hypothesis, inhibition of a region related to motor planning in both CD patients and healthy controls enlarged prefrontal and parietal involvement, suggesting that impaired movement planning is indeed compensated by the increased input of more distant prefrontal and parietal cortical regions. [6], [48], [49] Recruitment of these areas is also seen in learning new or complex movements, necessitating increased attention, integration of multimodal information, and working memory processes. [43] We suggest that the connectivity increase of areas within the ECN represents a new state of the dystonic brain, which came about by neuroplastic changes driven by this chronic movement disorder. Alternatively, the suggested compensatory effect could be due to more direct online control of dystonic contractions during the MRI-scan. But then one would expect to find that the observed connectivity changes within the ECN are modified by BoNT treatment because this significantly decreased dystonic posturing, which we did not.

Rather than a compensatory role, the strengthened connectivity in the ECN could also be a more primary abnormality in CD. A more provocative hypothesis would be that excessive attentional control in CD leads to faulty motor planning and execution. In the development of motor acquisition, attention is first focused at the separate motor elements of the motor behaviour. When the motor program is consolidated, the focus shifts to the consequences in order to optimize motor performance. [50] Recently, it was suggested that, in dystonia, disproportionate attention to these internal mechanisms of motor planning could lead to increased control of movement, distorting motor representations. Because motor representations are optimally made implicitly, this could in fact worsen motor performance. [51] This alternative hypothesis is supported by the occurrence of dystonia after heightened attention or increased arousal. [52].

### Exploratory RSN Analysis – Primary Visual Network

The PVN originates from the occipital pole. Within this network *reduced* connectivity in CD patients was demonstrated in the PFC, PMC, SPL, middle temporal gyrus, and to lesser extent the primary sensorimotor cortex and early visual cortex. After BoNT treatment, there was partial restoration of these dystonia-related connectivity differences, as we observed an increase of connectivity for the early visual cortex and primary motor cortex.

The PVN is concerned with visual perception and processing of spatial information in relation to several aspects of spatial cognition, e.g. spatial memory, perception, and orientation. [53]–[56] It is the source of three different pathways extending to the PFC, PMC and temporal areas, all of which displayed abnormal levels of connectivity with the PVN. This cluster of regions and its pathways play a central role in spatial cognition, exerting both an indirect and direct influence on motor output. These areas serve the construction of a representation of space, in which spatial body knowledge is integrated from visual, proprioceptive and somatosensory information, all crucial for accurate movement. [39] In order to direct spatial movement and subsequently adjust motor parameters, this egocentric reference frame is translated in premotor-, prefrontal-, and temporal-parietal connections. [39], [57], [58] Loss of connectivity between areas involved in this process, as observed here for CD patients, may thus be a substrate for deficient spatial cognition, implying that patients experience difficulties with spatial movements, manifesting for example as abnormal head posture or movements. Further evidence for faulty spatial cognition and deficient integration of body-related knowledge is provided by the mental rotation deficits in CD patients [58] and poor performance in several tasks of visuospatial function. [59] Consequently, CD patients adopt an allocentric frame of reference to direct their movements, which was previously confirmed during various spatial perception and memory tasks. [60], [61].

### Treatment-related Connectivity

Connectivity with the SMN and PVN demonstrated post-treatment alterations in regions associated with motor planning and spatial (visual) processing. In accordance with the results presented here, cortical reorganization after BoNT treatment was previously reported in focal dystonia. [62], [63] Also, premotor and early visual areas were previously found to exhibit post-BoNT altered activity patterns moving towards the patterns found in healthy controls. [64], [65] BoNT is known to act through both alpha and gamma motor endings altering muscle spindle afferent input. [66] The central effect of BoNT is, thus, proposed to be indirect. This change in sensory feedback from the treated dystonic muscles may lead to cortical reorganization as a neuroplastic response to peripheral sensory alterations. [67] The post-treatment re-coupling of motor planning-related regions further supports our hypothesis of deficient motor planning as a key defect in the pathophysiology of CD. Although we expected to observe a correlation between treatment-related connectivity and the reduction in dystonia severity as an expression of altered peripheral feedback, the absence of this correlation should not be used as argument to exclude the existence of an indirect and peripheral effect of BoNT on cortical reorganization nor suggest a direct and central effect of BoNT. It is more likely that we did not pick up this correlation because of the sample size and the rather narrow range in the reduction of dystonia severity. The finding of altered connectivity in the visual cortex after BoNT was only found for t = 1> t = 0. It remains to be seen whether this can be confirmed by future studies. A BoNT-related effect in the ventral premotor cortex was found contra-lateral for t = 1> t = 2 in contrast to the primary contrast t = 1> t = 0, most probably due to a threshold effect.

### Interpretational Issues

In the interpretation of the results, it has to be taken into account that we refer to differences in functional connectivity, i.e. the temporal correlation between neurophysiological events that are spatially remote, as assessed by independent component analysis and dual regression. This connectivity measure does not necessarily imply a direct connection between the studied cerebral areas. In addition to co-variations of regions A and B, also region C can be the driver of the observed differences in connectivity. Nevertheless, we are looking at regions where the A/B relationship on the basis of anatomy is well understood. Thus while there is a technical limitation in the present study, we also need to place findings into the context of known anatomical connections.

In this study, we have not measured possible dystonic activity of the cervical musculature during scanning. However, it is known that in most CD patients dystonic posturing is absent or minimal in the supine position. Even more, non of the subjects reported dystonic posturing during scanning in the post-scanning debriefing. However, this limitation has to be taken into account when interpreting the results.

In the classic view, dystonia is thought to be derived from dysfunctional basal ganglia. There is indeed plenty of data that point to abnormal basal ganglia function, including some neuroimaging studies. [16], [68]–[72] Still, the exact role of the basal ganglia in the abnormal motor system physiology of the dystonic brain (see introduction) remains incompletely understood. In correspondence to this traditional view, we found differential connectivity between the right mid-posterior putamen and left FPN, but the effect did not survive correction for multiple comparisons. This lack of robust proof of basal ganglia involvement may, however, be due to technical limitations. Because of their deep location and the use of a surface head coil during data acquisition, the signal intensity of the basal ganglia is much lower than that of the cortex. Also, some substructures within the basal ganglia are small and the precise location can vary between subjects. Therefore, while we appear to have sufficient power to detect significant (corrected) neocortical effects, we might suffer from a lack of statistical power in order to detect subcortical modulations in resting functional connectivity. Appreciating these technical limitations, the fact that we found some suggestion of abnormal cortico-striatal connectivity, and the known anatomically and functionally relations between the basal ganglia and the RSNs investigated here, might still indicate that the basal ganglia circuitry is, directly or more remotely, pathologically involved in CD. A more provocative inference based on the lack of significant basal ganglia involvement may be that the abnormal motor system physiology observed in CD primarily reflects a dysfunction of cortical areas with normally operating basal ganglia (a ‘garbage in/garbage out’ line of thinking). Further exploration of basal ganglia connectivity changes is thus needed.

### Conclusion

In CD, selected regions in the PFC, PMC and SPL, i.e. regions related to motor planning, exhibited reduced connectivity with regard to the sensorimotor network, a distributed network that comprises the PMC, SM1, and secondary somatosensory cortex. Selected regions in the PFC, PMC, SPL, and middle temporal gyrus areas, i.e. regions related to spatial cognition, demonstrated reduced connecticity with regard to the primary visual network, a network originating from the occipital cortex. We hypothesize that the abnormalities in these two resting state networks indicate a primary deficit of motor planning and disturbed spatial cognition in CD, which is supported by the finding of BoNT-induced re-coupling between some of these areas and these two networks. The observation of increased connectivity of regions in the PFC, PMC, parts of the primary sensorimotor cortex and early visual cortex with regard to the executive control network, a network that comprises regions in the frontal cortex and parietal cortex, is supposedly explained by compensatory reorganization in order to reduce the consequences of the motor planning defect inflicted by the other network abnormalities, but could also be a primary trait.

## Supporting Information

Figure S1 **Altered connectivity within the ECN and SMN.** Depicted here are the between-group effects for two RSNs. Between-group effects are corrected for family-wise errors (p≤0.05 for **A.** and **B.**). **A.** shows brain regions linked to the *ECN*, exhibiting *in*creased connectivity in the CD group. **B.** shows an assembly of regions abnormally connected to the *SMN*, demonstrating *de*creased connectivity within the CD group. ^*^The right column (green) shows the original RSNs used in the dual regression approach, thresholded at Z = 2,0. These are PICA spatial maps of healthy subjects derived from Smith et al. (Smith et al., 2009) Images are t-statistics overlaid on the MNI-152 standard brain. The left hemisphere of the brain corresponds to the right side in this image.
(DOC)

Table S1 **Patient details.** F = female; M = Male; No. = number; TWSTRS = Toronto Western Spasmodic Torticollis Rating Scale; X = only MRI scan at t = 0.^ 1^Mean used for age and age at onset; median used for TWSTRS scores. The results for TWSTRS scores ranged from 6 to 22 (mean 18.4) before injection (t = 0) and from 1 to 17 (mean 10.4) after injection (t = 1), demonstrating a significant improvement due to BoNT (TWSTRS, Z = −4.11, p = .00). The mean duration botulinum toxin treatment was 7.6 years.
(DOC)

Table S2 **Local maxima of regions with altered connectivity within the SMN.** C = controls, IPC/hIP = intraparietal cortex, OP = operculum, P = patients, RSN = resting state network, SMA = supplementary motor area. Between-group effects are corrected for family-wise errors (p≤0.05).
(DOC)

Table S3 **Local maxima of regions with altered connectivity within the ECN.** C = controls, OP = operculum, P = patients, RSN = resting state network, SMA = supplementary motor area. Between-group effects are corrected for family-wise errors (p≤0.05).
(DOC)
