# Supplementary material for: Task-Free Functional MRI in Cervical Dystonia Reveals Multi-Network Changes That Partially Normalize with Botulinum Toxin
Source: PLoS One. 2013 May 1;8(5):e62877. doi: 10.1371/journal.pone.0062877 (PMC3641096; doi:10.1371/journal.pone.0062877)
Supplement: Figure S1 — Altered connectivity within the ECN and SMN. Depicted here are the between-group effects for two RSNs. Between-group effects are corrected for family-wise errors (p≤0.05 for A. and B.). A. shows brain regions linked to the ECN, exhibiting increased connectivity in the CD group. B. shows an assembly of regions abnormally connected to the SMN, demonstrating decreased connectivity within the CD group. *The right column (green) shows the original RSNs used in the dual regression approach, thresholded at Z = 2,0. These are PICA spatial maps of healthy subjects derived from Smith et al. (Smith et al., 2009) Images are t-statistics overlaid on the MNI-152 standard brain. The left hemisphere of the brain corresponds to the right side in this image. (DOC) [file pone.0062877.s001.doc]

**Figure S1. Altered connectivity within the ECN and SMN.**


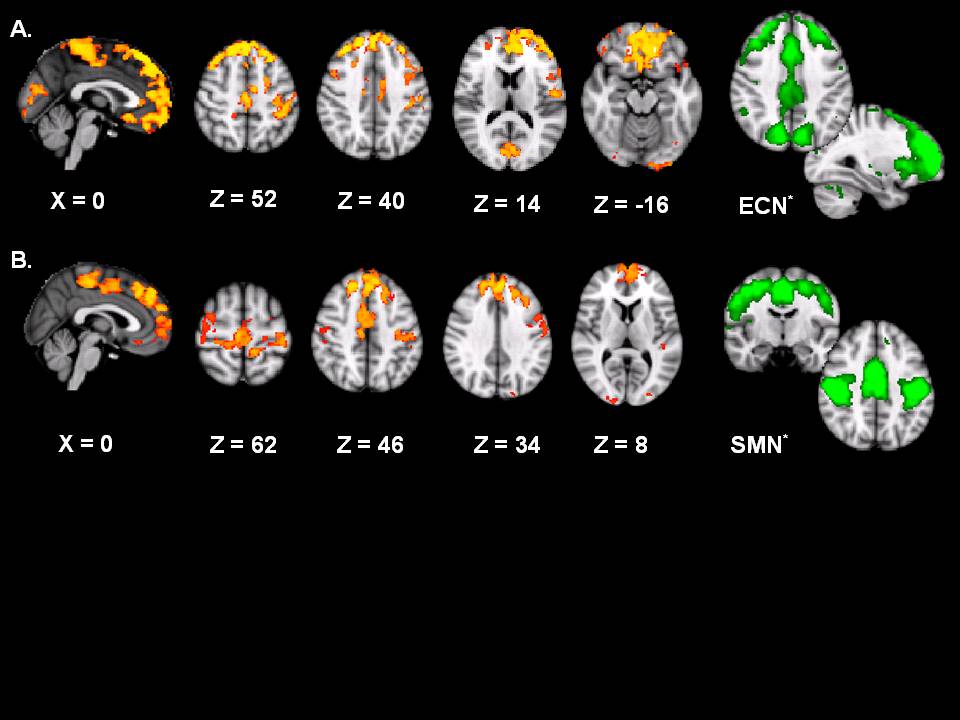


Depicted here are the between-group effects for two RSNs. Between-group effects are corrected for family-wise errors (p ≤ 0.05 for **A.** and **B.**). **A.** shows brain regions linked to the *ECN*, exhibiting *in*creased connectivity in the CD group. **B.** shows an assembly of regions abnormally connected to the *SMN*, demonstrating *de*creased connectivity within the CD group. ^*^The right column (green) shows the original RSNs used in the dual regression approach, thresholded at Z=2,0. These are PICA spatial maps of healthy subjects derived from Smith et al. (Smith et al., 2009) Images are t-statistics overlaid on the MNI-152 standard brain. The left hemisphere of the brain corresponds to the right side in this image.
