# Supplementary material for: Task-Free Functional MRI in Cervical Dystonia Reveals Multi-Network Changes That Partially Normalize with Botulinum Toxin
Source: PLoS One. 2013 May 1;8(5):e62877. doi: 10.1371/journal.pone.0062877 (PMC3641096; doi:10.1371/journal.pone.0062877)
Supplement: Table S1 — Patient details. F = female; M = Male; No. = number; TWSTRS = Toronto Western Spasmodic Torticollis Rating Scale; X = only MRI scan at t = 0. 1Mean used for age and age at onset; median used for TWSTRS scores. The results for TWSTRS scores ranged from 6 to 22 (mean 18.4) before injection (t = 0) and from 1 to 17 (mean 10.4) after injection (t = 1), demonstrating a significant improvement due to BoNT (TWSTRS, Z = −4.11, p = .00). The mean duration botulinum toxin treatment was 7.6 years. (DOC) [file pone.0062877.s002.doc]

**Table S**1. Patient details

| **Patient No.** | **Sex** | **Age**  **(years)** | **Duration symptoms** | **TWSTRS** |  |  |
| --- | --- | --- | --- | --- | --- | --- |
|  |  |  |  | **t=0** | **t=1** | **t=2** |
| **1** | M | 46 | 2 | 6 | 1 | 8 |
| **2** | M | 45 | 25 | 21 | 8 | 20 |
| **3** | F | 53 | 15 | 15 | 12 | 20 |
| **4** | F | 58 | 2 | 14 | 8 | 20 |
| **5** | F | 60 | 20 | 19 | 13 | 20 |
| **6** | F | 58 | 10 | 17 | 9 | 20 |
| **7** | F | 48 | 15 | 20 | 14 | 19 |
| **8** | M | 40 | 20 | 22 | 17 | 22 |
| **9** | F | 82 | 22 | 17 | 12 | 19 |
| **10** | F | 63 | 9 | 19 | 15 | 20 |
| **11** | F | 60 | 3 | 17 | 7 | 17 |
| **12** | F | 50 | 21 | 19 | 9 | 17 |
| **13** | M | 57 | 18 | 17 | 3 | 20 |
| **14** | F | 71 | 5 | 20 | 3 | 17 |
| **15** | F | 61 | 9 | 20 | 9 | 19 |
| **16** | M | 50 | 3 | 21 | 14 | 21 |
| **17** | M | 60 | 4 | 19 | 10 | 18 |
| **18** | F | 69 | 10 | 19 | 15 | 20 |
| **19** | M | 47 | 8 | 22 | 14 | 22 |
| **20** | F | 49 | 12 | 18 | X | X |
| **21** | M | 67 | 16 | 20 | 12 | 20 |
| **22** | M | 59 | 20 | 20 | 16 | 20 |
| **23** | F | 64 | 16 | 20 | 8 | 19 |
| **Mean (SD)**  **or**  **median^1^** | F | 60.4 (9.3) | 12.8 (6.9) | 19 | 9 | 19 |
|  | M | 52.3 (8.8) | 12.6 (8.1) | 20 | 12 | 20 |
|  | Total | 57.3 (9.8) | 12.7 (72) | 19 | 11 | 19 |

F = female; M = Male; No. = number; TWSTRS = Toronto Western Spasmodic Torticollis Rating Scale; X = only MRI scan at t=0. ^1^Mean used for age and age at onset; median used for TWSTRS scores.

The results for TWSTRS scores ranged from 6 to 22 (mean 18.4) before injection (t=0) and from 1 to 17 (mean 10.4) after injection (t=1), demonstrating a significant improvement due to BoNT (TWSTRS, Z = -4.11, p=.00). The mean duration botulinum toxin treatment was 7.6 years.
