# Supplementary material for: Task-Free Functional MRI in Cervical Dystonia Reveals Multi-Network Changes That Partially Normalize with Botulinum Toxin
Source: PLoS One. 2013 May 1;8(5):e62877. doi: 10.1371/journal.pone.0062877 (PMC3641096; doi:10.1371/journal.pone.0062877)
Supplement: Table S2 — Local maxima of regions with altered connectivity within the SMN. C = controls, IPC/hIP = intraparietal cortex, OP = operculum, P = patients, RSN = resting state network, SMA = supplementary motor area. Between-group effects are corrected for family-wise errors (p≤0.05). (DOC) [file pone.0062877.s003.doc]

**Table S2. Local maxima of regions with altered connectivity within** the SMN

| **Contrast** | **RSN** | **Region** | **Area** | **Side** | **X** | **Y** | **Z** | **p-value** |
| --- | --- | --- | --- | --- | --- | --- | --- | --- |
| **Sensori-motor network** | **C > P_t=0_** | Superior frontal gyrus | 8/9 | Left | -2 | 46 | 36 | 0.005 |
|  |  |  | 9 | Right | 6 | 62 | 22 | 0.012 |
|  |  | Middle frontal gyrus | 6/8 | Left | -28 | 20 | 48 | 0.047 |
|  |  |  | 44 | Left | -48 | 12 | 38 | 0.033 |
|  |  |  | 6 | Left | -30 | -6 | -64 | 0.048 |
|  |  | Inferior frontal gyrus | 45 | Left | -48 | 42 | 0 | 0.038 |
|  |  | Superior temporal gyrus | OP1/ TE1.1 | Left | -46 | -28 | 12 | 0.039 |
|  |  | Superior medial gyrus | 8/9 | Right | 14 | 44 | 38 | 0.008 |
|  |  |  | 9/10 | Right | 4 | 66 | 8 | 0.024 |
|  |  | Anterior cingulate/paracingulate | 25/32 | Right | 6 | 46 | 28 | 0.012 |
|  |  | Middle cingulate cortex | 6 | Left | -8 | 0 | 44 | 0.029 |
|  |  | Precentral gyrus | 6 | Right | 36 | -16 | 66 | 0.037 |
|  |  |  | 4p | Right | 40 | -14 | 42 | 0.045 |
|  |  | SMA | 6 | Right | 4 | -24 | 54 | 0.008 |
|  |  |  |  | Left | 0 | -4 | 58 | 0.026 |
|  |  | Mid orbital gyrus | 11 | Right | 6 | 60 | -4 | 0.024 |
|  |  | Postcentral gyrus | 4a/p | Right | 28 | -30 | 60 | 0.026 |
|  |  |  | 1 | Right | 44 | -28 | 60 | 0.041 |
|  |  |  | IPC | Right | 48 | -28 | 44 | 0.047 |
|  |  |  | 3b/2 | Left | -42 | -32 | 58 | 0.028 |
|  |  | Inferior parietal lobule | IPC/ hIP2 | Left | -48 | -34 | 42 | 0.048 |
|  |  | Precuneus | 5M | Left | -4 | -42 | 56 | 0.012 |
|  |  | Cuneus | 18 | Right | 20 | -98 | 10 | 0.038 |
|  |  | Superior occipital gyrus | 7A | Left | -16 | -84 | 42 | 0.036 |
|  |  | Middle occipital gyrus | 18 | Left | -18 | -90 | 14 | 0.036 |

C = controls, IPC/hIP = intraparietal cortex, OP = operculum, P = patients, RSN = resting state network, SMA = supplementary motor area. Between-group effects are corrected for family-wise errors (p ≤ 0.05)
