# Supplementary material for: Task-Free Functional MRI in Cervical Dystonia Reveals Multi-Network Changes That Partially Normalize with Botulinum Toxin
Source: PLoS One. 2013 May 1;8(5):e62877. doi: 10.1371/journal.pone.0062877 (PMC3641096; doi:10.1371/journal.pone.0062877)
Supplement: Table S3 — Local maxima of regions with altered connectivity within the ECN. C = controls, OP = operculum, P = patients, RSN = resting state network, SMA = supplementary motor area. Between-group effects are corrected for family-wise errors (p≤0.05). (DOC) [file pone.0062877.s004.doc]

Table S3. Local maxima of regions with altered connectivity within the ECN

| **RSN** | **Contrast** | **Region** | **Area** | **Side** | **X** | **Y** | **Z** | **p-value** |
| --- | --- | --- | --- | --- | --- | --- | --- | --- |
| **Executive control network** | **P_t=0_ > C** | Superior frontal gyrus | 6 | Right | 16 | 34 | 56 | 0.004 |
|  |  |  | 8/9 |  | 18 | 44 | 42 | 0.004 |
|  |  |  | 6/8 |  | 20 | 24 | 42 | 0.019 |
|  |  |  | 6 | Left | -14 | 28 | 56 | 0.004 |
|  |  |  | 9/10 |  | -20 | 60 | 6 | 0.006 |
|  |  |  | 6 |  | -22 | 0 | 56 | 0.018 |
|  |  | Middle frontal gyrus | 8/9 | Right | 36 | 36 | 36 | 0.004 |
|  |  |  | 10 |  | 28 | 46 | 14 | 0.022 |
|  |  |  | 10 | Left | -28 | 44 | 12 | 0.022 |
|  |  |  | 44 |  | -46 | 16 | 36 | 0.022 |
|  |  | Inferior frontal gyrus | 44/45 | Right | 52 | 36 | -16 | 0.037 |
|  |  |  |  | Left | -44 | 18 | -18 | 0.038 |
|  |  | Superior medial gyrus | 8 | Left | 0 | 32 | 54 | 0.003 |
|  |  | Insula | 13/14 | Left | -36 | -2 | -2 | 0.038 |
|  |  | Rectal gyrus | 11 | Left | -10 | 46 | -16 | 0.010 |
|  |  | Mid orbital gyrus | 11 | Right | 6 | 56 | -2 | 0.006 |
|  |  | Middle temporal gyrus | 21/22 | Left | -62 | -18 | 0 | 0.024 |
|  |  | Precentral gyrus | 44 | Right | 50 | 8 | 42 | 0.022 |
|  |  |  | 6/4a | Left | -40 | -8 | 48 | 0.046 |
|  |  | Paracentral lobule | 3a | Right | 16 | -40 | 52 | 0.038 |
|  |  |  | 4a |  | 10 | -30 | 78 | 0.044 |
|  |  |  | 4/6 | Left | -6 | -32 | 61 | 0.005 |
|  |  | Anterior cingulate cortex | 25/32 | Right | 4 | 44 | 12 | 0.006 |
|  |  |  |  | Left | -8 | 44 | 12 | 0.005 |
|  |  | Middle cingulate cortex | 6 | Left | 0 | -14 | 44 | 0.006 |
|  |  | Superior parietal lobule | 5L | Right | 16 | -46 | 66 | 0.020 |
|  |  | Postcentral gyrus | 2/3b | Left | -44 | -26 | 48 | 0.006 |
|  |  |  | OP4 |  | -54 | -10 | 14 | 0.010 |
|  |  |  | 4p/3a | Right | 14 | -36 | 60 | 0.018 |
|  |  | Precuneus | 5M | Left | -14 | -48 | 56 | 0.039 |
|  |  | Lingual gyrus | V3 | Right | 40 | -90 | -12 | 0.029 |
|  |  |  |  | Left | -12 | -58 | -4 | 0.047 |
|  |  | Calcarine gyrus | 18 | Right | 10 | -80 | 14 | 0.014 |
|  |  |  | 17 | Left | -6 | -84 | 6 | 0.010 |
|  |  | Cuneus | 18 | Left | 4 | -84 | 20 | 0.010 |

C = controls, OP = operculum, P = patients, RSN = resting state network, SMA = supplementary motor area. Between-group effects are corrected for family-wise errors (p ≤ 0.05).
